# The Honey Bee Parasite *Nosema ceranae*: Transmissible via Food Exchange?

**DOI:** 10.1371/journal.pone.0043319

**Published:** 2012-08-16

**Authors:** Michael L. Smith

**Affiliations:** Bees@WUR, Plant Research International, Wageningen University, Wageningen, The Netherlands; Sheffield University, United States of America

## Abstract

*Nosema ceranae*, a newly introduced parasite of the honey bee, *Apis mellifera*, is contributing to worldwide colony losses. Other *Nosema* species, such as *N. apis*, tend to be associated with increased defecation and spread via a fecal-oral pathway, but because *N. ceranae* does not induce defecation, it may instead be spread via an oral-oral pathway. Cages that separated older infected bees from young uninfected bees were used to test whether *N. ceranae* can be spread during food exchange. When cages were separated by one screen, food could be passed between the older bees and the young bees, but when separated by two screens, food could not be passed between the two cages. Young uninfected bees were also kept isolated in cages, as a solitary control. After 4 days of exposure to the older bees, and 10 days to incubate infections, young bees were more likely to be infected in the 1-Screen Test treatment vs. the 2-Screen Test treatment (*P* = 0.0097). Young bees fed by older bees showed a 13-fold increase in mean infection level relative to young bees not fed by older bees (1-Screen Test 40.8%; 2-Screen Test 3.4%; Solo Control 2.8%). Although fecal-oral transmission is still possible in this experimental design, oral-oral infectivity could help explain the rapid spread of *N. ceranae* worldwide.

## Introduction

*Nosema ceranae* is a parasitic microsporidium that until the 1990s infected only the Asian honey bee, *Apis cerana*. It was first reported in the European honey bee, *Apis mellifera*, in Spain in 2006 [1], though later it was found in archived bee samples in the U.S. dating back to 1995 [2], and in Uruguay pre-1990 [3]. Evidently, *N. ceranae* spread rapidly through *A. mellifera* worldwide, initially unbeknownst to researchers or beekeepers, and with variable reports of its virulence [4], [5], [6]. When honey bee populations experienced unprecedented die-offs between 2003–2006, dubbed Colony Collapse Disorder, *N. ceranae*, which had just been discovered to infect bees, was identified as a possible causative agent, whether acting alone [7], or in conjunction with other infections [8].

Before 2006, it was thought that *A. mellifera* was infected by only one microsporidian parasite, *Nosema apis*, a relatively benign pathogen [9]. Infectious spores of *N. apis* leave one host individual via the feces and enter the next host individual through the mouthparts (a fecal-oral pathway) [9]. *N. apis* infections are correlated with colonies exhibiting diarrhea [9], which could enhance transmission. In contrast, *N. ceranae* is not associated with diarrhea – and yet it has spread rapidly, even replacing *N. apis* throughout much of the world [10], [11], [6]. It may be that *N. ceranae* has an alternate pathway for transmission.

Ingested *Nosema* spores pass through their host’s digestive tract until the spores germinate, the polar filament punctures epithelial cells, and they replicate within [12], [13]. In infected bees, spore counts in the midgut can exceed 10^7^ [14]. Filtering hairs in the bee’s proventriculus act as a one-way valve, preventing spores in the midgut from reaching the crop [15]. These hairs are capable of retaining particles as small as 0.5 µm in diameter [16]; *N. ceranae* spores measure 4.4×2.2 µm [17]. While this suggests that most *N. ceranae* spores do not pass from the midgut into the crop, there is a possibility that some spores could cross this barrier. *N. ceranae* spores have been found in the pollen loads of bees, which include some regurgitated nectar from the crop [17]. If spores are able to pass the proventriculus, and reach the crop, they could be regurgitated to other colony members during food exchange, providing a mechanism for oral-oral transmission.

To determine whether *N. ceranae* can be transmitted during food exchange, experiments were conducted using hoarding cages. Initially uninfected bees were separated from infected bees using one screen or two screens. In the 1-Screen cages, food could be passed between infected and uninfected bees, but not in the 2-Screen cages. These cages reduce the likelihood of fecal-oral transmission, but cannot entirely eliminate the fecal-oral pathway. Infection rates in the initially uninfected bees were determined post-treatment. If oral transmission occurs, then post-treatment infection rates would be predicted to be higher in 1-Screen separated bees than in 2-Screen separated bees.

## Methods

Experiments were performed at the Bee Lab of Plant Research International, Wageningen University, The Netherlands. 5 trials were performed starting 19 April 2011, and 9 trials starting 11 May 2011.

### Treatments

**Figure 1:**
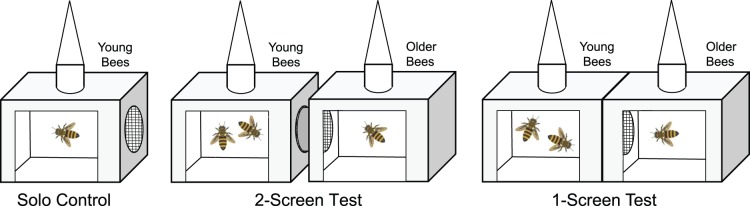
Diagram of cages used in the three treatments. Triangles above cages are feeders, and the circles on the sides represent the screening between cages. The 2∶1 ratio of bees in the image represents the ratio of older bees to young bees (labeled on top of each cage). Multiple bees were kept in each cage, ranging between 10 and 70 bees. In the 2-Screen Test, but not in the 1-Screen Test, there was spacing between paired cages to prevent food exchange between bees in the two cages.

The experiment involved giving groups of young, initially uninfected bees three treatments. In the Solo Control, young bees were placed in a cage by themselves. In the 2-Screen Test, young bees were placed in one cage, and older infected bees were placed in an adjacent cage; the two groups were separated by 1.5 mm mesh screens spaced 0.6 cm apart for the 19 April trials, and 1.0 cm apart for the 11 May trials. In the 1-Screen Test, young bees were placed in one cage, and older infected bees were placed in an adjacent cage; the two groups were separated by only one screen (see Figure 1).

In all three treatments, the young bees were caged for 14 days. They were fed *ad libitum* with a 50% sucrose solution (w/w) feeder. Over the 14-day test period, any dead bees were removed from the cages every other day. Mortality rates did not differ between the three treatments (Young bees- ANOVA; *F* = 0.39, df between groups = 2, df within groups = 11, *P* = 0.69, Older bees- ANOVA; *F* = 0.15, df between groups = 1, df within groups = 8, *P* = 0.71). Dead bees were not included in the data set. Each young bee that survived for 14 days was euthanized in a −80°C freezer, before dissecting its midgut to determine if it was positive or negative for *N. ceranae* spores. Bees not dissected immediately were stored in a −20°C freezer.

In both the 2-Screen Test and 1-Screen Test, older bees were installed in the adjacent cage from day 1 to 4 at an approximate 2∶1 ratio (older bees: young bees; mortality altered the final ratios, as shown in Table 1). The older bees were fed *ad libitum* with a 50% sucrose solution (w/w) feeder. Only older bees that survived the full 4 days were included in the data set. Midgut dissection occurred post −80°C euthanasia. After the older bees were removed, the cage that held them was left attached to the cage holding the young bees, to minimize handling time and control for airborne exposure between the two cages.

**Table 1:** Summary of results from the cage trials.

Start Date	Hive #	Treatment	ScreenDistance (cm)	No. ofolder bees	No. of deadolder bees	% older bees infected	No. ofyoung bees	No. of deadyoung bees	% young bees infected	Average % young bees infected ±1 SD
19-Apr	6	Solo Control	–	–	–	–	6	8	0.0	2.8±5.6
11-May	2	Solo Control	–	–	–	–	11	9	0.0	
11-May	3	Solo Control	–	–	–	–	18	25	11.1	
11-May	6	Solo Control	–	–	–	–	19	8	0.0	
19-Apr	2	2-Screen Test	0.6	23	17	100.0	16	4	6.3	3.4±3.1
19-Apr	4	2-Screen Test	0.6	19	21	100.0	18	2	5.6	
11-May	2	2-Screen Test	1.0	49	1	4.1	20	5	0.0	
11-May	3	2-Screen Test	1.0	67	3	16.4	14	21	0.0	
11-May	6	2-Screen Test	1.0	60	0	8.3	20	10	5.0	
19-Apr	2	1-Screen Test	0	25	15	92.0	21	0	33.3	40.8±31.1
19-Apr	4	1-Screen Test	0	23	17	95.6	19	1	89.5	
11-May	2	1-Screen Test	0	49	1	6.1	20	5	5.0	
11-May	3	1-Screen Test	0	59	21	23.7	15	26	46.7	
11-May	6	1-Screen Test	0	60	0	8.3	27	5	29.6	

In the 1-Screen Test treatment, the food was removed from the young bees from day 2–4 (three days total). This forced them to receive food from the older bees. The young bees were observed to receive food via trophallaxis from the older bees, and in both the 1-Screen and 2-Screen treatments, both the older bees and the young bees were often found congregating on the screen. Mortality in the young bees while they did not have access to food was no different than during the rest of the treatment period (data not shown).

### Bees

Honey bees (*Apis mellifera* L.) were taken from four colonies in the Wageningen research apiary. In the 2-Screen Test and 1-Screen Test treatments, the older infected bees and the young bees in adjacent cages came from the same colony.

#### Young bees

Recently emerged bees (easily recognized by their ‘fuzzy’ and ‘wet’ appearance) were taken directly from combs using a plastic tube aspirator. They were held in a 250 mL plastic container until they were placed in a test cage. To confirm that only young bees had been collected, they were exposed to white light while placing them in the test cages in the climate room; if some older bees had been collected accidentally, they would have flown in the white light. No bees flew.

#### Older bees

Bees of unknown ages (but no recently emerged bees, based on the criteria discussed above) were brushed off combs and placed into a 250 mL plastic container. The older bees were then installed in a 40×40×50 cm flight cage with wire screen walls inside the climate room, with a 50% sucrose solution (w/w) *ad libitum* feeder, for 2 hours (in the April 19 trials) or overnight (in the May 11 trials). This enabled the older bees to defecate before they were placed in the test cages, and many of them did defecate. After the opportunity for cleansing flights, the older bees were collected with an aspirator and placed in test cages. Bees were carefully removed from the flight cage to prevent them from contaminating the test cages with feces, but there was no way to prevent bees from defecating once inside a test cage, which was observed. Defecation within the test cages did not occur on the screen facing the young bees, and tissue paper on the floor of the cage absorbed feces and kept them from becoming spread in the cage. Because of these precautions, infection from a contaminated screen is less likely, though still possible.

#### Infecting bees

To ensure high infection rates in the older bees, marked bees were artificially infected for the 19 April trials. Young bees were taken directly from the frame and brought into the climate room where they were marked with paint according to hive number, placed in a plastic cage (20×20×20 cm), and starved for 1 hour. They were then sprayed with 50% sucrose solution (w/w) containing *N. ceranae* spores collected from dissected midguts of infected bees. The solution concentration made ∼400,000 spores available per bee, depending on rates of ingestion. Spore count measured using a haemocytometer. As the bees cleaned themselves they imbibed the spore-laden solution. The bees were kept in the climate room overnight with an *ad libitum* 50% sucrose feeder (w/w), and were then returned to their original colony. After 12 days, the marked bees were collected with an aspirator, and treated as older bees (described above).

In the May 11 trials, bees were not artificially infected. Infections in the older bees were dependent on natural infections within each colony, and varied between cages. This was done to mimic naturally occurring infections, as well as a range of infection rates.

### Identifying Bees Infected with *N. ceranae*

All bees surviving until the end of the trial were individually dissected. Each bee’s midgut was placed onto a glass slide by pulling apart the two anterior tergites with tweezers and cutting out the midgut. Tweezers and micro-scissors were rinsed and dried after each dissection. A DMLB Leica microscope, at 400x magnification, was used to determine if each midgut was positive or negative for *N. ceranae* spores. A positive sample had many spores, >100’s, seen in multiple field-of-views. To be sure that all samples were adequately examined for spores, at least 25 field-of-views per slide were examined. Since bees infected with *N. ceranae* regularly produce up to 10^7^ spores, positive and negative samples were easily distinguishable. If a sample only had a few spores (∼2–10 spores total), it was marked as uninfected, presumably due to contamination from the forceps. The forceps were immediately changed. This conservative approach perhaps underestimated the infection rates in both the young and older bees, but was preferable to overestimating the infection rates.

### Cages

Individual cages measured 11×7.5×5 cm and were made of cardboard with transparent plastic sheet on one side. A hole was cut in the top of each cage to fit a plastic feeder holding ∼25 mL of 50% sucrose solution (w/w in water). Tissue paper was placed on the bottom of each cage. There was a 4 cm-diameter hole on one side of each cage. This hole was covered with 1.5 mm-mesh screen, to keep the bees from escaping their cages, and to expose the young bees to the older bees. All cages were kept on metal grate shelves in a walk-in climate room (25°C with 50% relative humidity). The room could be illuminated with white or red light.

In the Solo Control treatment, the cages were kept separate. In the 2-Screen Test treatment, cages were arranged in pairs with their screens facing each other. Cardboard spacers kept the two screens at a fixed distance throughout the experiment (0.6 cm for the April 19 trials, 1.0 cm for the May 11 trials). These distances are longer than a honey bee’s proboscis (∼2 mm); so bees could not share food between the cages. The cages were taped together to prevent movement. In the 1-Screen Test treatment, the two cages were placed in direct contact with the 4 cm-holes facing each other, and only one screen between the two cages. Bees could share food through the screen. All cages were discarded after use. For cage diagrams, see Figure 1.

### PCR Detection

Real-Time PCR was used to confirm spore species identity. Spore suspensions were tested for both *N. apis* and *N. ceranae*, using primers from Bourgeois et al. [18]. All microsporidia samples were positive for *N. ceranae* and negative for *N. apis*. For a more detailed description of the PCR methods, please see Methods S1.

### Statistical Analysis

Data were analyzed using statistical software R v2.10.1 (The R Foundation for Statistical Computing). The dependent variable, Percent Infected Young, was subjected to a square root transformation to provide variances suitable for statistical analysis using an ANOVA. To compare the three treatments, a Tukey HSD Test was performed.

## Results

**Figure 2:**
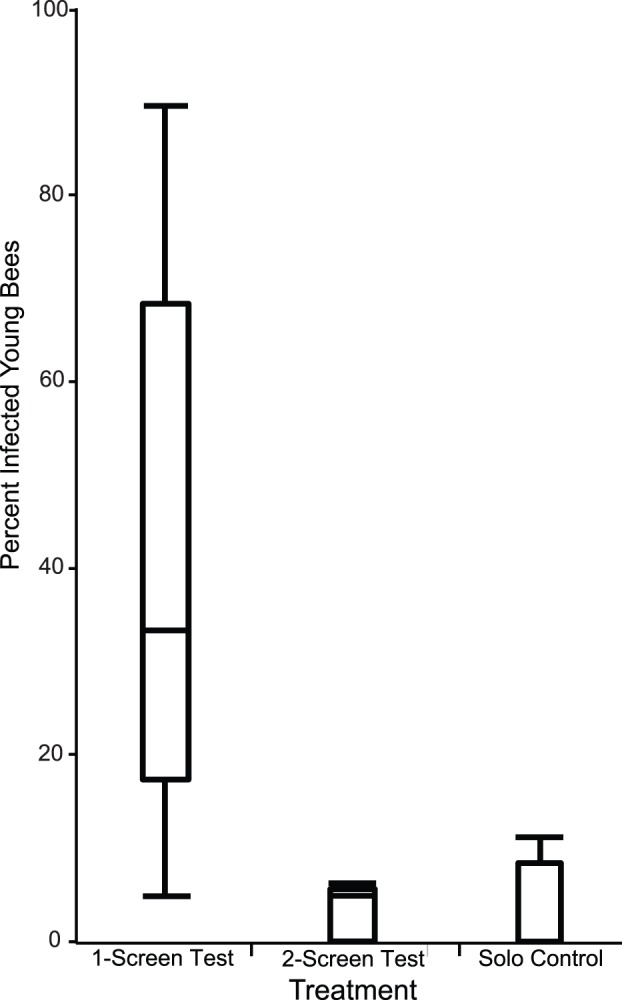
Percent infected young bees per treatment. Box and whisker plot comparing the percent of young bees that became infected throughout all cage trials for the three treatments.

Table 1 summarizes the results of all 14 trials. There was a higher mean percentage of infected young bees in the 1-Screen Test trials (40.8±31.1%) than in the Solo Control and the 2-Screen Test trials (2.8±5.6% and 3.4±3.1%, respectively). The distributions of the percent infected young bees for the three treatments are shown in Figure 2. There are significant differences in percent infected young bees among the three treatments (ANOVA; *F* = 9.70, df between groups = 2, df within groups = 11, *P* = 0.0037). The percent infected young bees is significantly higher for the 1-Screen Test treatment relative to the 2-Screen Test and to the Solo Control (Tukey HSD; *P* = 0.0097 and 0.0065, respectively). The percentages of infected young bees for the 2-Screen Test and Solo Control are not significantly different (Tukey HSD; *P* = 0.90).

One hive was used twice as a source of bees in each treatment (see Table 1), so there is a small risk of pseudo-replication. To check that pseudo-replication did not skew the statistical analysis, one of the trials was removed (the one from May 11) in each treatment where bees came from the same hive, and the statistical analysis was repeated. The percentages of infected young bees in the three treatments were still significantly different (ANOVA; *F* = 13.8, df between groups = 2, df within groups = 8, *P* = 0.0025), the 1-Screen Test was still significantly different from the 2-Screen Test and the Solo Control (Tukey HSD; *P* = 0.0058 and 0.0044, respectively), and the 2-Screen Test and Solo Control were not significantly different (Tukey HSD; *P* = 0.86).

## Discussion

These results are consistent with the hypothesis that *N. ceranae* can be spread when infected bees feed uninfected bees. Young uninfected bees that were fed by older infected bees (fed through a single screen) developed *N. ceranae* infections at a level 13-times higher than young uninfected bees unable to feed from older infected bees (separated by two screens). This supports the oral-oral transmission hypothesis, whereby spores are passed across the single screen during food exchange, resulting in increased infection rates.

Although these results suggest that *N. ceranae* can be spread orally, the fecal-oral pathway could still be present in this experimental design. Since the older bees were kept in cages for 4 days, bees did defecate, despite preventative efforts (see methods). No feces were observed on the screen, but infective spores could have been defecated elsewhere. It is possible that spores could be passed from the forelegs to the proboscis, and then transmitted to other bees during food exchange. It seems unlikely, however, that such high infection rates arose from occasional spore relocation, since it relies on multiple chance events. Future experiments could further reduce the likelihood of fecal-oral transmission by keeping the older infected bees in their cages for a shorter period of time, to reduce defecation.

While oral transmission of *N. ceranae* has been previously suggested [19], this study provides the first experimental support. Whether spores pass from the midgut to the crop (through the proventriculus), or are produced directly in the feeding glands, is unknown. To date, there is no histological evidence for infective spores being produced outside of the midgut, even though *N. ceranae* DNA has been found in hypopharyngeal and salivary glands [20]. While it is known that the proventriculus filters spores from the crop within 5–20 minutes [15], it remains to be determined whether this filtering is 100% effective. Although speculative, it seems possible that very high spore counts in the midgut [14] may compromise the ability of the proventriculus to block spores from reaching the crop- the source of regurgitated food shared with nestmates. If *N. ceranae* spores can be transmitted orally, then this would explain its rapid spread, since food sharing occurs throughout a colony.

In the Solo Control and 2-Screen Test, low levels of infection were found, despite efforts to select young bees, which are unlikely to be infected. There are three possible explanations for this finding: (1) the young bees were not young bees. This seems unlikely, since the collected young bees did not fly when exposed to white light; older bees would have flown. (2) The young bees were already infected with spores, either from their first post-eclosion feeding [21], [22], or from fecal matter within the hive. This is possible, but unlikely, since nurse bees have the lowest *N. ceranae* infection rates [7], and *N. ceranae* does not cause diarrhea. (3) *N. ceranae* spores are infective via airborne fecal matter. The older bees did defecate in their cages, and dried fecal particles carrying spores could have entered all cages within the shared climate chamber. Of these explanations, (2) and (3) are the most plausible. Unfortunately, the current experimental design cannot distinguish between these two explanations for why a low level of infection was found in the Solo Control and 2-Screen Test.

In the 1-Screen Test, infection rates in the young bees ranged from 5% to 89%. This variation is expected, since the source of infection (percent infected older bees) varied between 6% and 96%. In the two trials with highly infected older bees (>90% infection), the young bees developed infection at 33.3% and 89.5%. Behavioral differences could explain this additional variation: a single infected older bee could efficiently spread the infection if it fed numerous young bees. *N. ceranae* infected bees have been shown to exhibit a higher level of hunger [12], which could make them less likely to share food. Of course, if spores can also be transmitted to the food provider, increased hunger would increase the spread of *N. ceranae*; a behavioral change that would increase the parasite’s transmission.

If *N. ceranae* is passed via food exchange, as these data suggest, but cannot confirm, then it could be an overlooked infective pathway for the disease. Oral transmission may also occur in *N. apis*, but previous work only focused on a fecal-oral pathway [9]. A comparative analysis of infectious pathways could demonstrate a change in infectious strategy between closely related microsporidia. These experiments should be repeated with *N. apis* to test whether it too can be spread orally. If the rates of infection in the 1-Screen Test treatment were reduced to the same level as seen in the 2-Screen Test and Solo Control, then that would provide strong evidence that *N. ceranae*, but not *N. apis*, is able to take advantage of oral-oral transmission.

## Supporting Information

Methods S1 **Detailed description of the PCR methods used for detecting** ***N. ceranae*** **and** ***N. apis*** **.**
(DOC)
