# Supplementary material for: The Honey Bee Parasite Nosema ceranae: Transmissible via Food Exchange?
Source: PLoS One. 2012 Aug 16;7(8):e43319. doi: 10.1371/journal.pone.0043319 (PMC3420906; doi:10.1371/journal.pone.0043319)
Supplement: Methods S1 — Detailed description of the PCR methods used for detecting N. ceranae and N. apis . (DOC) [file pone.0043319.s001.doc]

SUPPLEMENTARY MATERIAL:

**PCR Detection:**

Real-Time PCR was used to confirm spore species identity. Spores were collected from 20-40 foragers from all the colonies used in the experiment. Spore suspensions were made by dissecting the midguts of all the foragers, and macerating them with a pestle and mortar in dH2O. All spores used in the experiment were freshly harvested. Of 5-8 spore suspensions made, 4 spore suspensions were tested for *N. apis* and *N. ceranae*. The samples were first extracted for DNA, using a Masterpure complete DNA and RNA purification kit (MC85200 Epicentre, BiozymTC). Samples were then subjected to Real-Time PCR: once to test for *N. ceranae*, and once for *N. apis*. In both tests, the same forward primer was used (sequence: aagagtgagacctatcagctagttg), and reverse primer (sequence: ccgtctctcaggctccttctc), from **Bourgeois et al.** [18]. To detect *N. ceranae*, one LNA probe was used (sequence: ggctGtGacgggtaacg), and to detect *N. apis,* a different LNA probe was used (sequence: aaggcaAtAAcgggtaacgg). Both probes used FAM fluorophores. All primers and probes were purchased from Biolegio (Nijmegen, Netherlands). The manufacturers protocol was followed, using an AB 7500 Real-Time PCR System (Applied Biosystems, Foster City, USA). Samples were cycled for 40 reps: 95.0°C for 0:15, and 55.0°C for 0:45. Data were collected and analyzed using SDS 2.05 software (Applied Biosystems, Foster City, USA). All four of the spore samples were positive for *N. ceranae*, and negative for *N. apis*, at the standard threshold set by SDS software (ΔRn for *N. apis* = 0.01813, ΔRn for *N. ceranae* = 0.008868).

Probes were developed by Richard van Hoof, PRI, Wageningen University. All probes were checked for species specificity using Visual OMP Nucleic Acid Software (DNA Software, Ann Arbor, USA). To experimentally confirm species specificity of the probes, previously confirmed spore samples of *N. ceranae* and *N. apis* (provided by Reinhold Siede, Bee Institute of Kirchhain, Germany) as well as a double negative (dH_2_O) and a double positive (mixture of *N. apis* and *N. ceranae*) were tested with both probes. All reactions with *N. apis* spores were only positive in the presence of the *N. apis* probe, and all reactions with *N. ceranae* spores were only positive in the presence of the *N. ceranae* probe*.* There was no evidence of cross-reactions, false positives, or false negatives, at the standard thresholds set by the SDS software.
